# Feasibility of recruiting young adults with low socioeconomic status for formative evaluation of a smoking cessation mobile intervention

**DOI:** 10.18332/tpc/194164

**Published:** 2024-11-13

**Authors:** Michael Wakeman, Sherine El-Toukhy

**Affiliations:** 1 Division of Intramural Research, National Institute on Minority Health and Health Disparities, National Institutes of Health, Rockville, United States

**Keywords:** smoking cessation interventions, recruitment feasibility, mobile applications, young adults, low socioeconomic status

## Abstract

**INTRODUCTION:**

Participant recruitment is critical to the success of smoking cessation trials. However, recruitment feasibility studies for inclusion and exclusion criteria commonly used in smoking cessation research remain scarce. We assessed the feasibility of recruiting potential research volunteers (PRVs) under two sets of inclusion criteria to inform eligibility requirements for a smoking cessation mobile intervention trial.

**METHODS:**

We invited PRVs nationwide to participate in qualitative evaluation of a smoking cessation mobile application. To be eligible under Criteria I, participants were aged 18–29 years, neither four-year college graduates nor enrollees, exclusive cigarette smokers, willing to quit within 30 days, and not using cessation aids. Criteria II expanded eligibility to those using cigarettes and non-combustible tobacco products (e.g. e-cigarettes) and willing to quit within 6 months. We calculated recruitment yields and associated costs.

**RESULTS:**

Of 10533 PRVs screened for eligibility, 48 were enrolled. Only 54 (0.5%) participants qualified under Criteria I and 164 (1.6%) under Criteria II. Age ineligibility was the top reason for exclusion (66.7%), whereas lifetime smoking, quit timeframe, and other tobacco product use contributed to ineligibility rates ranging from 46.5% to 65.3%. Enrolled participants were equally split by sex and roughly reflected the racial/ethnic composition of the United States. American Indians, who have the highest smoking prevalence, were <5% of enrolled participants. Recruitment costs averaged $106 per PRV.

**CONCLUSIONS:**

Eligibility requirements used in cessation trials were restrictive for recruitment efforts. Relaxing inclusion criteria will reflect current tobacco use patterns and facilitate the timely completion of trials within budgetary thresholds.

## INTRODUCTION

Efficient participant recruitment is essential for the success of interventions trials, including smoking cessation interventions^1,2^. However, studies repeatedly fail to meet recruitment thresholds and often require long completion timeframes at high costs^3^. Specific to smoking cessation trials, evidence of recruitment challenges common to health intervention research exists such as higher ineligibility rates among racial and ethnic minorities (vs Whites)^4,5^.

Recruitment feasibility studies allow researchers to test and modify aspects of the research deemed infeasible or ineffective to facilitate the successful completion of large-scale effectiveness trials^1,2^. However, few studies exist on the effectiveness of recruitment strategies for populations that smoke^6^. There are even fewer studies on recruitment yields for commonly used inclusion and exclusion criteria in smoking cessation trials^5^. Examples of such requirements include nicotine dependence, smoking frequency and intensity, use of tobacco products and/or cessation aids^7-10^. Other eligibility requirements relate to the intervention delivery medium (e.g. mobile phone ownership/access) are commonly applied for technology-based smoking cessation interventions^11^. More importantly, limited evidence exists on the effects of eligibility requirements on the feasibility of recruiting populations disproportionally affected by smoking^3^.

To illustrate the feasibility of recruiting research participants for upcoming trials to evaluate a mobile smoking cessation application, we assessed if our eligibility requirements would allow recruitment of a sufficient number of research participants, allowing us to refine our eligibility requirements and estimate recruitment timeline and costs for future studies.

## METHODS

This is a recruitment feasibility study that was conducted from January to April 2020. We partnered with UserWorks (Silver Spring, MD), a user-experience design firm, to recruit potential research volunteers (PRVs). PRVs are candidates who are interested in a research study but are yet to be screened for their eligibility to participate in the study. UserWorks recruited PRVs through its research panels and commercial platforms (e.g. Craigslist). PRVs received an invitation email to participate in a qualitative study on the acceptability and usability of a smoking cessation mobile application.

Interested PRVs were screened by phone for eligibility under two sets of requirements. Under Criteria I, PRVs were eligible if they were aged 18–29 years, neither graduates nor enrollees in a four-year college as an indicator of low socioeconomic status (SES)^12^, exclusive cigarette smokers who smoked at least 100 cigarettes in their lifetime and were current smokers (i.e. smoked every day or some days), willing to quit within 30 days, not using cessation aids, smartphone owners, and spoke English. Under Criteria II, we broadened eligibility to include PRVs who used non-combustible tobacco products (e.g. e-cigarettes) alongside cigarettes and were willing to quit within 6 months. We aimed to have ≤60% of enrolled participants of the same sex and ≤70% of a single racial/ethnic group. All PRVs provided data on their sex, race/ethnicity, and age, after which the screening interview ceased once a participant was deemed ineligible. Thus, beyond basic demographics, data were collected from subsets of screened candidates who progressed through the screening questionnaire.

We calculated recruitment yields and associated costs under both eligibility criteria. We computed chi-square tests to examine differences in the distribution of demographic characteristics between eligible and ineligible PRVs. Analyses were completed in SPSS (IBM, Version 29.0.2.0).

The National Institutes of Health Institutional Review Board (IRB) deemed the study exempt on 11 October 2019. UserWorks obtained an exempt IRB decision from an external IRB on 19 November 2019 with an amendment approved on 26 February 2020.

## RESULTS

We screened 10533 PRVs over four months. There were 54 PRVs (0.5%) who qualified to enroll under the restricted Criteria I and 164 PRVs (1.6%) who qualified under the relaxed Criteria II (Table 1). Recruitment costs averaged $324 and $106 per eligible participant under Criteria I and 2, respectively.

**Table 1:** Recruitment yield by eligibility criteria over recruitment timeframe, January–April 2020 (N=10533)

	*Recruitment month*								*Total ineligible*			*Total eligible*	
	*January*		*February*		*March*		*April*						
	*n*	*%*	*n*	*%*	*n*	*%*	*n*	*%*	*n*	*% ^a^*	*Cum. %*	*n*	*% ^a^*
**Total screened**	3315	31.5	3846	36.5	3210	30.5	162	1.5					
**First reason for exclusion**													
Age	2617	37.3	2359	33.6	1973	28.1	74	1.1	7023	66.7	66.7	3510	33.3
Education level	220	16.2	565	41.7	538	39.7	32	2.4	1355	12.9	79.5	2155	20.5
Current school	99	15.0	265	40.2	275	41.7	20	3.0	659	6.3	85.8	1496	14.2
Lifetime smoking	144	20.7	307	44.2	226	32.5	18	2.6	695	6.6	92.4	801	7.6
Smoking frequency	44	24.9	80	45.2	51	28.8	2	1.1	177	1.7	94.1	624	5.9
Considering quit	67	40.4	68	41.0	29	17.5	2	1.2	166	1.6	95.7	458	4.3
**Restricted Criteria I**													
Quit timeframe	84	28.1	128	42.8	79	26.4	8	2.7	299	2.8	98.5	159	1.5
Other tobacco use	21	20.4	51	49.5	26	25.2	5	4.9	103	1.0	99.5	56	0.5
Cessation aids use	1	50.0	1	50.0	0	0	0	0	2	0.0	99.5	54	0.5
**Relaxed Criteria II**													
Quit timeframe	52	26.0	87	43.5	55	27.5	6	3.0	200	1.9	97.6	258	2.4
Other tobacco use	18	25.7	37	52.9	13	18.6	2	2.9	70	0.7	98.2	188	1.8
Cessation aids use	8	33.3	13	54.2	2	8.3	1	4.2	24	0.2	98.4	164	1.6

a Percent (in)eligible of 10533 total potential research volunteers screened. Cum.: cumulative.

Age accounted for the largest single decrease in eligibility (n=7023; 66.7%) followed by lifetime smoking (n= 695; 46.5%) and education level (n=1355; 38.6%) among PRVs who answered these questions (Supplementary file Table 1). Quitting timeframe and use of other tobacco products resulted in the exclusion of 65.3% (n=299) and 64.8% (n=103) of participants under Criteria I and of 43.7% (n=200) and 27.1% (n=70) under Criteria II, respectively. There were no differences between eligible and non-eligible PRVs in the distribution of race/ethnicity, education level, and smoking frequency (all p>0.05).

A total of 48 participants were enrolled in the qualitative study of our smoking cessation mobile intervention. Sex and ownership of Androids and iPhones was equally represented among enrolled participants. The racial/ethnic composition of enrolled participants largely reflected the US population.

## DISCUSSION

The recruitment rate of young adults with low SES who smoked cigarettes was 1.6%, much lower than eligibility rates reported in the literature^5^. Results were instructive in modifying eligibility requirements for future evaluation trials of our smoking cessation mobile intervention. Modifications include broadening eligibility requirements to being aged ≥18 years and non-exclusive cigarette smoker. Due to the importance of SES and race/ethnicity for cessation efforts^13^, we will maintain the requirement of having low SES and aim to diversify PRVs by race and ethnicity and sex in upcoming trials. This study exemplifies the importance of recruitment feasibility studies in determining the sufficiency of recruitment rates by eligibility criteria that would facilitate the successful and timely completion of large-scale trials and the generalizability of research findings.

The eligibility requirements assessed in this study proved too restrictive for recruitment efforts. Specifically, demographic composition (e.g. age) and smoking behaviors (e.g. smoking frequency) are common in cessation research. While this ensures the homogeneity amongst participants and reduces noise from confounding variables, such requirements may jeopardize the successful completion of large-scale trials at worst or extend study completion times and increase costs at best. For example, based on the monthly eligibility trends observed in this study, it would require ≥2 years to recruit 474 participants for a planned (2^3^) full factorial optimization experiment and we would have to screen 30442 PRVs at the 1.6% recruitment rate observed under Criteria II. Recruiting additional PRVs to account for attrition rates typical in research studies or to allow for subgroup analysis by sex and race/ethnicity often mandated by funding agencies would further lengthen recruitment timeframes and increase costs^3^. Extending trial completion times can introduce confounders (e.g. seasonality) and delay the translation of health interventions^14^.

It may be beneficial to forsake some exclusion criteria for others that advance the tobacco control and health disparities fields. For example, age-inclusive interventions could extend their benefits to all adults who smoke, particularly as smartphone ownership is about 90% across age groups under ≤64 years^15^. Similarly, eligibility requirements based on prominent smoking patterns remained consistent over time to ensure comparability across research studies. However, recruiting individuals who do not exclusively smoke cigarettes aligns with the changing tobacco landscape where cigarette smoking is declining, especially among youth, and e-cigarette, dual, and poly-tobacco use is rising^13^. Indeed, dual use of cigarettes and non-combustibles was the top tobacco use pattern among our PRVs (n=113; 43.8%). This would allow researchers to examine intervention effects on other outcomes (e.g. product switching) alongside smoking cessation and would support comprehensive interventions aimed at reducing nicotine use and dependence rather than focusing on single products. Advancements in mobile technologies allow for tailoring and personalizing intervention content, dose, and delivery, which negates the need for restrictive enrollment criteria^11^. Relaxing eligibility requirements would free resources to recruit populations among which tobacco use is highly prevalent^13^. For example, only 4.2% of enrolled participants were American Indian/Alaskan Native, despite having the highest smoking rates of all racial/ethnic groups^13^. More importantly, researchers should be encouraged to conduct recruitment feasibility studies and publish their data on recruitment and accrual rates according to standardized reporting guidelines^3^. These practices would highlight innovative recruitment methods and satisfy the need to shorten study duration while having adequate representation of target populations.^3^

### Limitations

Several limitations are noteworthy. Results reflect recruitment rates for different eligibility requirements. Eligibility numbers are based on the first reason of exclusion, which is dependent on the order of screening questions. This was done to avoid burdening participants who were not compensated for answering the screening questionnaire. Language proficiency (and other proxy questions such as citizenship status) was deemed sensitive for UserWorks’ research panels and was assessed subjectively by interviewers during the screening interview. We opted to use education level as an indicator of low SES to lower participant burden given the number of questions required to assess education level and the well-documented missingness on income questions^12^. Results reflect the recruitment avenues and methods employed by UserWorks. Partnerships with national research firms, smoking cessation clinics, and community organizations could have yielded higher recruitment rates and shorter timeframes. Due to the COVID-19 pandemic, we opted for nationwide recruitment for remote participation (rather than recruiting locally for in-person participation).

## CONCLUSIONS

Restrictive eligibility requirements produced low recruitment rates, prolonged recruitment timelines, and increased recruitment costs. Results were instructive in broadening the eligibility requirements for future trials of our smoking cessation mobile intervention. Results emphasize the importance of recruitment feasibility studies that assess eligibility requirements to ensure a successful and timely completion of large-scale intervention studies with adequate representation of populations most important for tobacco control efforts.

## Supplementary Material



## Data Availability

The data supporting this research are available from the authors on reasonable request.
