# Supplementary material for: Feasibility of recruiting young adults with low socioeconomic status for formative evaluation of a smoking cessation mobile intervention
Source: Tob Prev Cessat. 2024 Nov 13;10:10.18332/tpc/194164. doi: 10.18332/tpc/194164 (PMC11558696; doi:10.18332/tpc/194164)
Supplement: Supplementary file 1 [file TPC-10-53-s1.pdf]

Supplementary Table 1. Characteristics of research participants by eligibility criteria, January to April 2020 (N = 10533).

	Screened (N = 10533)		Eligible to enroll, criteria 1 (N = 54)			Eligible to enroll, criteria 2 (N = 164)			Enrolled (N = 48)	
	n	%	*n*	%	*p*	*n*	%	*p*	*n*	%
**Sex**					.265			.830		
Female	5749	54.6	33	61.1		85	51.8		23	47.9
Male	4557	43.3	21	38.9		79	48.2		25	52.1
Other	45	0.4	-	-		-	-		-	-
Missing	182	1.7	-	-		-	-		-	-
**Race and ethnicity**					.578			.532		
Hispanic or Latino	1538	14.6	4	7.4		20	12.2		6	12.50
NH American Indian, Alaska Native	145	1.4	5	9.3		6	3.7		2	4.2
NH Native Hawaiian, Pacific Islander	62	0.6	2	3.7		5	3.0		1	2.1
NH Asian American	861	8.2	1	1.9		5	3.0		2	4.2
NH Black or African American	1981	18.8	12	22.2		40	24.4		12	25.0
NH White	5626	53.4	28	51.9		84	51.2		24	50.0
NH Other and mixed race	171	1.6	2	3.7		3	1.8		1	2.1
Missing	149	1.4	0	0		1	0.6		0	0
**Age**					-[4]			-[4]		
≤17 years	139	1.3	-	-		-	-		-	-
18-29 years	3510	33.3	54	100.0		164	100.0		48	100.0
30-39 years	2761	26.2	-	-		-	-		-	-
40-49 years	1830	17.4	-	-		-	-		-	-
50-59 years	1490	14.1	-	-		-	-		-	-
≥60 years	781	7.4	-	-		-	-		-	-
Missing	22	0.2	-	-		-	-		-	-
**Highest educational degree**					.155			.065		
Less than high school	128	3.6	7	13.0		10	6.1		3	6.25
High school graduate	594	16.9	18	33.3		65	39.6		16	33.3
Vocational school or some college	1433	40.8	29	53.7		89	54.3		29	60.4
College graduate or postgraduate	1295	36.9	-	-		-	-		-	-
Missing	60	1.7	-	-		-	-		-	-
Ineligible to answer question	7023									
**Current school enrollment**					.586			**.017**		
Yes	976	45.3	7	13.0		23	14.0		5	10.4

No	1129	52.4	47	87.0		141	86.0		43	89.6
Don't know, prefer not to answer, missing	50	2.3	-	-		-	-		-	-
Ineligible to answer question	8378									
**School type**					.862			**0.44**		
High school	53	5.4	2	3.7		3	1.8		0	0.0
Technical, trade, or vocational school	56	5.7	0	0		2	1.2		1	2.1
2-year college	258	26.4	5	9.3		18	11.0		4	8.3
4-year college	603	61.8	-	-		-	-		-	-
Graduate school	5	0.5	-	-		-	-		-	-
Missing	1	0.1	-	-		-	-		-	-
Ineligible to answer question[1]	9557		47	87.0		141	86.0		43	89.6
**Lifetime cigarette smoking**					-[4]			-[4]		
Yes	801	53.5	54	100		164	100		48	100
No	656	43.9	-	-		-	-		-	-
Don't know, missing	39	2.6	-	-		-	-		-	-
Ineligible to answer question	9037									
**Smoking frequency**					.265			.948		
Every day	356	44.4	33	61.1		90	54.9		38	79.2
Smoke days	268	33.5	21	38.9		74	45.1		10	20.8
Not at all	173	21.6	-	-		-	-		-	-
Don't know, missing	4	0.5	-	-		-	-		-	-
Ineligible to answer question	9732									
**Considering quitting**					-[4]			-[4]		
Yes	458	73.4	54	100		164	100		48	100
No	42	6.7	-	-		-	-		-	-
Don't know	124	19.9	-	-		-	-		-	-
Ineligible to answer question	9909									
**Quitting time frame**					**.023**			.211		
Next 7 days	46	10.0	14	25.9		24	14.6		15	31.2
Next 30 days	113	24.7	40	74.1		84	51.2		26	54.2
Next 6 months	99	21.6	-	-		56	34.1		7	14.6
Next year	93	20.3	-	-		-	-		-	-
More than one year	18	3.9	-	-		-	-		-	-
Don't know, missing	89	19.4	-	-		-	-		-	-
Ineligible to answer question	10075									

**Other tobacco use[2]**					-[4]			.251		
Cigarettes only use	75	29.1	54	100		68	41.5		37	77.1
Combustibles only use	4	1.6	-	-		-	-		-	-
Non-combustibles only use	10	3.9	-	-		-	-		-	-
Combustibles and non-combustibles dual use	7	2.7	-	-		-	-		-	-
Cigarettes and combustibles dual use	6	2.3	-	-		-	-		-	-
Cigarettes and non-combustibles dual use	113	43.8	-	-		96	58.5		11	22.9
Poly use	41	15.9	-	-		-	-		-	-
Missing	2	0.8	-	-		-	-		-	-
Ineligible[3]	10275									
**Cessation aids use[2]**					-[4]			-4		
Trying to quit on your own	181	70.2	54	100		164	100		48	100
Using any tobacco or nicotine delivery system	12	4.7	-	-		-	-		-	-
Using any smoking cessation complementary or alternative methods	4	1.6	-	-		-	-		-	-
Using any smoking cessation medications	1	0.4	-	-		-	-		-	-
Receiving any smoking cessation counseling support	2	0.8	-	-		-	-		-	-
Multiple resources (excluding quitting on own)	21	8.1	-	-		-	-		-	-
Missing	37	14.3	-	-		-	-		-	-
Ineligible	10275									

Beyond sex, race and ethnicity, and age, eligibility screening was terminated after the first reason for exclusion was met. Accordingly, the number of ineligible participants who were not asked screening questions increased with the progression of eligibility questionnaire.

NH= Non-Hispanic.

Bolded cells reflect significant *p* values at <.05 level.

[1]Includes those who were not currently enrolled in school (*n* = 1129 for all screened participants, 47 for those eligible under criteria 1, and 141 for those eligible under criteria 2).

[2]Frequencies for total screened is 258 participants (i.e., those willing to quit within 6 months).

[3]Includes 299 participants not considering quitting beyond 30 days under criteria 1 and 200 participants not considering to quit beyond 6 months under criteria 2.

[4]Chi-square test is not computed when one variable is a constant.

© 2024 Wakeman M. and El-Toukhy S.
